# A proposed artificial intelligence-based real-time speech-to-text to sign language translator for South African official languages for the COVID-19 era and beyond: In pursuit of solutions for the hearing impaired

**DOI:** 10.4102/sajcd.v69i2.915

**Published:** 2022-08-19

**Authors:** Milka Madahana, Katijah Khoza-Shangase, Nomfundo Moroe, Daniel Mayombo, Otis Nyandoro, John Ekoru

**Affiliations:** 1 School of Electrical and Information Engineering, University of the Witwatersrand, Johannesburg, South Africa; 2 Department of Audiology, School of Human and Community Development, University of the Witwatersrand, Johannesburg, South Africa

**Keywords:** artificial intelligence, COVID-19, hearing impaired, machine learning, speech, South Africa, text, translation, sign language

## Abstract

**Background:**

The emergence of the coronavirus disease 2019 (COVID-19) pandemic has resulted in communication being heightened as one of the critical aspects in the implementation of interventions. Delays in the relaying of vital information by policymakers have the potential to be detrimental, especially for the hearing impaired.

**Objectives:**

This study aims to conduct a scoping review on the application of artificial intelligence (AI) for real-time speech-to-text to sign language translation and consequently propose an AI-based real-time translation solution for South African languages from speech-to-text to sign language.

**Methods:**

Electronic bibliographic databases including ScienceDirect, PubMed, Scopus, MEDLINE and ProQuest were searched to identify peer-reviewed publications published in English between 2019 and 2021 that provided evidence on AI-based real-time speech-to-text to sign language translation as a solution for the hearing impaired. This review was done as a precursor to the proposed real-time South African translator.

**Results:**

The review revealed a dearth of evidence on the adoption and/or maximisation of AI and machine learning (ML) as possible solutions for the hearing impaired. There is a clear lag in clinical utilisation and investigation of these technological advances, particularly in the African continent.

**Conclusion:**

Assistive technology that caters specifically for the South African community is essential to ensuring a two-way communication between individuals who can hear clearly and individuals with hearing impairments, thus the proposed solution presented in this article.

## Introduction

The World Health Organization (WHO) projected that approximately 2.5 billion people globally will have some degree of hearing loss by 2050, and rehabilitation will be required for 700 million individuals (WHO, 2021a). Currently, over 430 million people in the world are in need of rehabilitation for hearing impairment (WHO, 2021a). Hearing impairment can range from a mild to profound hearing loss (Jorgensen, Benson, & McCreery, 2018) and can be unilateral or bilateral, thus leading to an individual having challenges in hearing loud sounds or conversational speech (WHO, 2021a). Individuals whose hearing loss ranges from mild to severe are usually referred to as hard-of-hearing individuals (Xie, Potměšil, & Peters, 2014). These individuals communicate using spoken language and, in most cases, may use hearing aids, cochlear implants or any other assistive devices and captioning (WHO, 2021a; Xie et al., 2014). When hearing loss in an individual is neglected, it can negatively impact various aspects of an individual’s life, including their communication needs (GBD 2019 Hearing Loss Collaborators, 2021; Russ, Tremblay, Halfon, & Davis, 2018). Irrespective of age, hearing impairment affects the psychosocial well-being, quality of life, interpersonal communication and economic independence of an affected individual (Joubert & Botha, 2019; Khoza-Shangase, 2019; Maluleke, Khoza-Shangase, & Kanji, 2021; Olusanya, Neumann, & Saunders, 2014). Among children, hearing impairment slows down progress in speech and language development, thus restricting their educational growth through limited career choices and vocational growth (Casoojee, Kanji, & Khoza-Shangase, 2021; Olusanya, et al., 2014). Children with hearing impairments are at an increased risk of emotional, sexual, physical and social abuse, and in worst-case scenarios, they may be murdered (Lomas & Johnson, 2012; Sebald, 2008). In adults, hearing difficulties may result in occupational stress, comparatively low earnings, abuse, prejudice, embarrassment, stigmatisation, difficulties in relating to family members and those close to them, social isolation, loneliness, depression and psychiatric disturbances (Khoza-Shangase, Moroe, & Edwards 2020; Moroe, Khoza-Shangase, Madahana, & Nyandoro, 2019; Mousley & Chaudoit, 2018; Olusanya et al., 2014).

Basic functional auditory abilities are significant for navigating daily activities at home, work, business and in social contexts (Dobie & Van Hemel, 2004). Sound identification, localisation, detection and recognition are made possible by audition. Another important role played by auditory abilities is the ability to perceive and comprehend spoken language (Dobie & Van Hemel, 2004). Hearing is, therefore, one of the essential senses that plays a significant role in communication. Challenges in hearing can jeopardise the communication process and influence the quality of life and well-being of an individual (Ramma & Sebothoma, 2016). Therefore, hearing is essential for survival, including accessing health awareness campaigns. Considering the significance of audition for survival, it is therefore imperative that individuals with hearing impairments, where possible, be provided with assistive technology; for example, the use of captions can be included when information is being passed via the television.

Currently, the world is navigating a pandemic caused by coronavirus disease 2019 (COVID-19), an infectious disease caused by the novel coronavirus (Perez, Perez, & Roman, 2020). Regulations and guidelines have been implemented in most countries to curb the spread of the disease (WHO, 2021b). In South Africa, some of the regulations were and are not limited to isolation for individuals infected or exposed to the COVID-19 virus, varying levels of lockdown, mandatory wearing of face coverings in public spaces for all individuals aged seven and above, as well as maintaining a distance of at least one and half metres from each other (South African Government, 2021) – with these regulations being adapted regularly based on the infection rate in the population. While physical distancing strategies and wearing of face coverings are important to slowing down of the spread of the disease, they have yielded unintended consequences such as the isolation of individuals who are hard of hearing, as some of them may use lip reading when they cannot hear someone properly (Homans & Vroegop, 2021; Mckinney, 2020). Face masks are worn to cover the lower part of the face, specifically, the nose and mouth. In relation to speech, face masks act as low-pass filters, and they attenuate medium to high frequencies of an individual’s speech (Goldin, Weinstein, & Shiman, 2020). Recent studies indicate that for N95 respirators and surgical masks, sound reduction ranges from 3 dB to 12 dB (Atcherson et al., 2017; Corey, Jones, & Singer, 2020; Goldin et al., 2020; Wolfe et al., 2020). This, therefore, significantly decreases speech recognition for all individuals, particularly so for individuals with hearing loss (Brotto et al., 2021; Rahne, Fröhlich, Plontke, & Wagner, 2021). Fiorella, Cavallaro, Di Nicola and Quaranta (2021) maintained that the propagation of sound waves is altered when an individual speaks while wearing a mask, with certain spectral components of the acoustic energy signal being filtered or attenuated. The masks are reported to also alter consonant intelligibility, and discrimination of unfamiliar speech sounds is also compromised (Corey et al., 2020; Fiorella et al., 2021). Furthermore, masks obstruct facial expressions and lip movements, which are critical sources of nonverbal communication (Brotto et al., 2021; Homans & Vroegop, 2021). Additionally, face masks are reported to impact on the content of the information being communicated (Saunders, Jackson, & Visram, 2021). Clear face masks have been presented as a solution for this dilemma (McKee, Moran, & Zazove, 2020); however, the usage and access to clear face masks varies from one individual to another, with uncontrollable adherence in varied social settings. Studies conducted in South Africa indicate that individuals with hearing impairment who need to lip read in order to understand found it very difficult to communicate with others while they are wearing masks (Mckinney, 2020).

Considering the pressure that COVID-19 currently places on health care, economic and social structures, it is imperative that all stakeholders have an ability to communicate swiftly and accurately during this pandemic (Sahni & Sharma, 2020). In this global public health crisis, efficient dissemination of knowledge results in prompt awareness and preparedness, which results in a less burdened health care system and less risk for health care workers (Weiner, Balasubramaniam, Shah, & Javier, 2020). The information should be disseminated very swiftly at a rate that is faster than the spreading epidemic (Sahni & Sharma, 2020). There is a need for the information disseminated to be reliable and accurate. Sahni and Sharma (2020) believed that this would result in quick implementation of policies; thus, compliance with prevention measures would lead to the number of cases of infection significantly declining. Delays in relaying of vital information by policymakers are detrimental to preventive measures.

Globally (Tavanai, Rouhbakhsh, & Roghani, 2021) and in South Africa (Adigun, Vivekanantharasa, & Obosu, 2021; Mckinney, 2020), hearing-impaired individuals have faced various challenges in accessing news and information on the COVID-19 outbreak, as well as in accessing educational and health awareness material, which heightened their psychological trauma (Adigun et al., 2021). Coronavirus disease 2019 seems to have further widened the already existing gap with regard to access to health-related information and health care services (Adigun et al., 2021). The lack of access to health care services by the deaf has been previously attributed to limitations in understanding and use of English-language vocabulary, limited use of sign language interpreters, infrequent contact with health care providers and the lack of adequate knowledge of various medical terms (Adigun et al., 2021; Kritzinger, 2011; McKinney, McKinney, & Swartz, 2020). In South Africa, some individuals with hearing impairment have limited access to advanced technology, and mostly rely on television or radio to access information on COVID-19. The South African Department of Health provided information about COVID-19, its transmission and prevention through the media which included the use of television, radio, media briefings and social media. Individuals who are deaf or with various levels of hearing impairment were excluded from accessing this information because some of the media briefings did not include sign language interpretation, there was a lack of closed captioning and some of the information provided was not accessible to individuals who are hard of hearing but at the same time do not have the literacy level that allows them to be able to read and understand the subtitles (Mckinney, 2020). Some television broadcasters increased access by ensuring that sign language interpreters were available, but this was only for main news bulletins and official briefings and not in the majority of other shows where crucial information was being shared. Moreover, sign language interpreters only facilitated access to the deaf and not the hearing impaired, who still use spoken and not South African sign language. Innovative technology (e.g. real-time captioning of live news) can be used, and this can expand access to the hearing impaired; however, currently available technology only caters for populations that can speak in English. The fact that real-time captioning can only be done in English in a linguistically diverse South African context, where English was reported to be spoken as a home language by less than 10% of the population (Alexander, 2021), is a challenge requiring a solution, hence the importance of the proposed artificial intelligence (AI)-based real-time speech-to-text to sign language translator for South African official languages.

Globally, COVID-19 news is constantly being updated, and rapid access to information is essential; however, individuals with hearing impairment challenges have struggled to keep up with news on television and on the radio. Anecdotally, students with hearing impairment have also struggled with the online live lecture experience, which was the forced teaching method adopted by most educational institutions following the implementation of COVID-19 regulations globally.

Prior to the onset of the COVID-19 pandemic, hearing loss was already linked to isolation for individuals who are deaf and hard of hearing (Park, 2020). The COVID-19 prevention policies and public health recommendations have disproportionately affected the social well-being of individuals. Meanwhile, COVID-19 has increased and highlighted existing inequalities for individuals with hearing impairment (Park, 2020). The literacy rate and numeracy rate of deaf learners have been historically known to be low. In South Africa, the average age of deaf adults who have attended schools for the deaf is lower than the international average, which is said to be at fourth grade level (Ng’ethe, Blake, & Glaser, 2015). In addition to that, apartheid caused racial inequalities in education development among the deaf, resulting in varying levels of literacy across different groups (Ng’ethe et al., 2015). Development of an end-to-end South African translator would facilitate two-way communication among individuals who can hear and those who cannot hear. Application of AI approaches is one of the effective solutions that have been previously employed in other nations to design such tools.

Artificial intelligence refers to a machine’s capacity to imitate aspects of human intelligence, with the objective to create machines that are capable of using the characteristics of human intelligence to problem-solve and adapt to an ever-changing environment (Sennott, Akagi, Lee, & Rhodes, 2019). Artificial intelligence has its roots in various fields, including mathematics, philosophy, psychology, neuroscience, linguistics, economics and computer engineering. Recently, AI has been used in disability research (Domingo, 2021; Sennott et al., 2019). Concepts associated with AI include machine learning (ML) and natural language processing (Sennott et al., 2019). Machine learning is a type or form of AI that enables machines to learn without being explicitly programmed (Domingo, 2021). Machine learning can be used in speech recognition to sense, interpret and facilitate new ways of assisting people with disability to access communication more readily (Sennott et al., 2019), and these are documented to already be in regular use in high-income countries (HICs). In automatic speech recognition (ASR), the raw speech is preprocessed, important features are extracted and ML is applied in recognising speech. Application of AI and ML in feature extraction and classification has been observed to have an accuracy of 99.01% (Adeyanju, Bello, & Adegboye, 2021).

Low- and middle-income countries (LMICs) like South Africa need to explore advances in technology that is currently available in HICs that cater for the hearing impaired. Considering the current paradigm shift in South Africa and the transition towards the Fourth Industrial Revolution (4IR), it is imperative that current technologies being developed in South Africa include AI and ML concepts in their design of solutions that are targeted towards bridging communication between the normally hearing and the hearing impaired. Alexander (2021) reported that English is only spoken by less than 10% of the South African population. The rest of the population uses their home languages to communicate, which includes isiZulu, South Africa’s most widely spoken language, used by almost a quarter (23%) of the population, isiXhosa (spoken by 16%), Afrikaans (13.5%), Sesotho sa Leboa (Sepedi) (9%), Setswana and Sesotho (both 8%), Xitsonga (4.5%), siSwati and Tshivenda (both 2.5%) and isiNdebele (2%). Translation of speech-to-text in other South African languages over and above English is therefore essential to ensuring effective communication with the entire linguistically and culturally diverse South African population during this COVID-19 pandemic era and beyond.

Hearing assistive technologies (for instance, frequency modulation and loop systems, telecommunication devices, sign language interpretation, alerting devices and captioning services) have been developed to assist individuals who are hard of hearing (Crandell & Smaldino, 1999; Kricos, 2007; McPherson, 2014; Van Leeuwen et al., 2021). With the well-documented limited access to such technologies for large numbers in LMICs, lip-reading and sign language remain key to communication access for the hearing impaired and the deaf. Furthermore, real-time captioning has only been developed for English-speaking individuals, and sometimes, depending on one’s accent, the translation of English speech-to-text may also not be accurate. Therefore, this article proposes the development of an AI-based real-time speech-to-text to sign language translator for South African languages following a scoping review. This translator can be used during and beyond the COVID-19 pandemic era. This research work forms part of a series of investigations that consider the end-to-end translation of speech-to-text or speech-to-text to sign language, or speech-to-sign-language using the AI approach. Each stage of the translation has real-life applications to individuals with varying levels of hearing impairment. For example, the speech to speech-to-text sign language will be mainly used by individuals whose level of literacy allows them to read and type. The speech-to-sign-language could be used for individuals who may not have a level of literacy that allows them to read and type but they understand sign language. The end-to-end translation will ensure that communication is two-way between individuals that can hear and those that cannot hear.

## Aims and objectives

The main aim of this study is to identify the gaps that exist in South Africa in the application of AI and ML techniques for the translation of speech-to-text to sign language, with the following specific objectives:

- To identify peer-reviewed publications published in English between 2019 and 2021 to explore evidence available that apply AI and ML techniques for the real-time translation of speech-to-text to sign language as a solution for the hearing impaired in South Africa.
- To propose the real-time translator of speech-to-text to sign language for South African official languages, which applies AI and ML techniques that can be used by hearing-impaired individuals who still use spoken and not South African sign language.

## Methodology

Adhering to Levac, Colquhoun, and O’Brien (2010) methodology, the researchers came to an agreement on a broad research question that was the focus of the scoping review and on the global study protocol, including specification of MeSH terms terms/keywords/phrases and selection of databases to be searched. For this scoping review, the Arksey and O’Malley’s (2005) framework was adopted, with the broad question that guided the current scoping review being, ‘What evidence is available on artificial intelligence-based real-time speech-to-text to sign language as a solution for the hearing impaired?’ This question was explored as a preliminary step towards the development of the proposed speech-to-text to sign language translator.

The search was conducted in December 2021 in the following five electronic databases: Scopus, Medline, ScienceDirect, PubMed and ProQuest. Only studies published in English between 2019 and 2021 (COVID-19 era) were included. The search consisted of the following terms: AI, speech-to-text to sign language, communication with hearing impaired, translation for hearing impaired.

**FIGURE 1:**
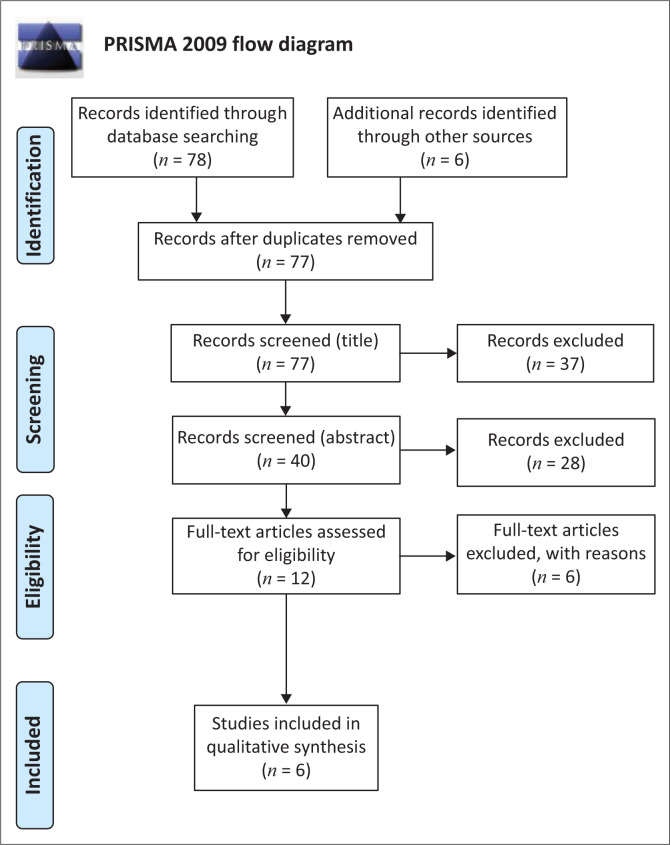
The Preferred Reporting Items for Systematic Reviews and Meta-Analyses flow diagram describing the process of study selection.

Initially, a total of 84 publications were identified for potential inclusion in this study. During data collation and organisation, seven studies were deleted as they were duplicates; therefore, only 77 studies were then considered. Of the 77, 37 were removed based on the titles and/or abstracts that were deemed as not meeting the focus of the study. Subsequently, 40 studies were evaluated for eligibility, and from these, 28 studies were excluded as they did not meet the study’s inclusion criteria. During the full-text review, a further six manuscripts were excluded, thus leaving six manuscripts for analysis (see Figure 1).

### Ethical considerations

This article followed all ethical standards for research without direct contact with human or animal subjects.

## Results and discussion

As depicted in Table 1, the review yielded six studies: Ezhumalai, Raj Kumar, Rahul, Vimalanathan and Yuvaraj (2021), Papastratis, Chatzikonstantinou, Konstantinidis, Dimitropoulos and Daras (2021), Harkude, Namade, Patil and Morey (2020), Shinde and Dandona (2020), Baumgärtner, Jauss, Maucher and Zimmermann (2020) and Shezi and Ade-Ibijola (2020). Thematic analysis revealed that of these studies, three were conducted in India (Ezhumalai et al., 2021; Harkude et al., 2020; Shinde & Dandona, 2020), one in Germany (Baumgärtner et al., 2020) and one in South Africa (Shezi & Ade-Ibijola, 2020). The remaining one was a literature review study conducted by Papastratis et al. (2021), thus not specific to one country. It is noteworthy that three studies were conducted in India. India, like South Africa, is classified as an LMIC, with limited resources and high incidence of hearing impairment.

**TABLE 1:** Summary of studies included in the scoping review documenting evidence on AI-based real-time speech-to-text to sign language as a solution for the hearing impaired during coronavirus disease 2019.

Author(s) & date	Publication title	Context or country	Aim	Technology	Conclusion	Recommendations
Ezhumalai et al. (2021)	Speech to sign language translator for hearing impaired	India	To design an application that converts the speech and text input into a sequence of sign language visuals. Speech recognition is used to convert the input audio to text, and it is further translated into sign language.	This system is designed to translate each word that is received as input into sign language. This project translates the words based on Indian sign language. Natural language processing – The filler words such as ‘is’, ‘are’, ‘was’, ‘were’, etc., are words that hardly contribute to the context in sign language conversion. Therefore, the system removes those filler words from the speech or sentence. Root words – The words may be in gerund form, plural form or adjective form. The proposed system will remove these forms of the words and find the root word from those words. These root words will be helpful in the effective conversion into sign language. Data set – The system has a large data set of Indian sign language words to map according to the text or text recognised from the speech. S, it will be helpful to all deaf people in India. It makes people understand most of the speech or text.	Speech to sign language translation is a necessity in the modern era of online communication for hearing-impaired people. It will bridge the communication gap between normal and hearing-impaired people.	The future work is to develop a chat application incorporated with this sign language translation system. This can be used in team meeting applications, where a live translator feature can be added to the application. Also, a sign language to text translating option can be added to this application.
Papastratis et al. (2021)	Artificial intelligence technologies for sign language	-	To provide a comprehensive review of state-of-the-art methods in sign language capturing, recognition, translation and representation, pinpointing their advantages and limitations.	Systematic literature search: Sign language recognition Continuous sign language recognition Isolated sign language recognition Sign language translation Sign language representation Realistic avatars Sign language production Applications	Most existing works deal with sign language recognition, while sign language capturing and translation methods are still not thoroughly explored. There is still room for improvement for applications, especially mobile ones, that can assist the deaf community.	Improvements can still be achieved in the accuracy of sign language recognition and production systems. Advances should be made in the extraction of robust skeletal features, especially in the presence of occlusions, as well as in the realism of avatars. Finally, it is crucial to develop fast and robust sign language applications that can be integrated in the everyday life of hearing-impaired people and facilitate their communication with other people and services.
Harkude et al. (2020)	Audio to sign language translation for deaf people	India	To develop a communication system for deaf people.	**Audio to text conversion:** Audio input is taken using python PyAudio module. Conversion of audio to text using microphone. Dependency parser is used for analysing the grammar of the sentence and obtaining the relationship between words.	Sign language translator is very useful in various areas – schools, colleges, hospitals, universities, airports, courts, anywhere anyone can use this system for understanding sign language to communicate. It makes communication between a normal-hearing person and a hard-of-hearing person easier.	The future work is to develop an application where the news channels can use it while giving news; in one corner of the screen, sign language will be displayed for deaf people. Right now only DD news is using this kind of presentation, but they are using a human being showing signs according to the speech of the person giving news live. So this will be a better idea that can be given to news channels. We look forward to expand the project by also including facial expressions into the system.
				Text to sign language: Speech recognition using Google Speech application programming interface (API). Text pre-processing using NLP. Dictionary-based machine translation. ISL generator: ISL of input sentence using ISL grammar rules. Generation of sign language with signing avatar.		
Baumgärtner et al. (2020)	Automated sign language translation: the role of artificial intelligence now and in the future	-	To develop systems for automated sign language recognition and generation.	Two kinds of approaches generate avatar animations: motion capturing (human movements are tracked and mapped to an avatar) keyframe animations (the entire animation is computer-generated).	The mentioned approaches have potential. In the future, the mentioned technologies can enable sign language users to access personal assistants, to use text-based systems, to search sign language video content and to use automated real-time translation when human interpreters are not available. With AI, automated sign language translation systems could help break down communication barriers for DHH individuals.	Taking this concept further, a daily life application based on smartphone technologies could be developed and automatically translate speech to sign language and vice versa. A range of (spoken and signed) languages could be supported, and the signer might additionally be able to choose or individualise the signing avatar.
Shezi and Ade-Ibijola (2020)	Deaf Chat: A speech-to-text communication aid for hearing DEFICIENCY	South Africa	To introduce a model and a tool (Deaf Chat) to communicate with hearing-impaired individuals based on artificial intelligence.	Deaf Chat uses speaker diarisation techniques to recognise and classify speakers before sending converted speech to text from one user to another. The Android studio integrated development environment (IDE) was used to develop the tool, and the International Business Machine (IBM) Corporation Watson API was used to build the application.	This paper presented a new model and a software prototype for facilitating communication with hearing-impaired individuals. This tool can be used to communicate with all three types of hearing-impaired individuals and further contributes to improving the lives of the targeted users. Deaf Chat is one of the first South African AI tools that were developed for social good, and it is perceived that it will open more opportunities for innovative research and development for this particular purpose.	Implement the model and design in an iOS and web-based version of Deaf Chat. Other additional features include quick access to emergency services for hearing-impaired users.
Shinde and Dandona (2020)	Two-way sign language converter for speech impaired	India	To propose a system that enables a two-way conversation between the speech impaired and other vocal individuals.	Phase-I: Converting a stream of input hand gestures to their relevant semantic text as well as audio output in real time. Phase-II: Converting an audio input to text and eventually displaying the relevant hand gesture images.	The prototype successfully demonstrates a solution to bridge the communication gap and recognises 320 words and converts them to hand gestures with 100% accuracy. It is also capable of breaking up sentences and displaying appropriate hand gestures of keywords in the sentence.	-

AI, Artificial intelligence; DHH, deaf or hard of hearing; NLP, natural language processing; ISL, Indian Sign Language.

Granular analysis of articles included in this review revealed that four studies were at conceptualisation stages – sign language recognition (continuous sign language recognition and isolated sign language recognition) or sign language representation (using realistic avatars) (Ezhumalai et al., 2021; Harkude et al., 2020; Papastratis et al., 2021; Shinde & Dandona 2020). Only one study by Shezi and Ade-Ibijola (2020) was at implementation phase – application (Deaf Chat Application).

Current review findings exposed the noticeable lack of urgency in developing effective AI technology for communication that includes people who are hard of hearing. Furthermore, these findings indicate a significant dearth of evidence focusing on the real-time speech-to-text to sign language translation for hearing-impaired individuals. Such a lacuna in evidence is an indictment on those involved in ensuring access to communication by everyone, including those with barriers to communication. The dearth of studies indicates the lack of aligning clinical research with emerging technologies, as well as missed opportunities in maximising innovations that AI and ML have introduced – systems that can be trained to develop ASR to convert speech-to-text to sign language for people with communication difficulties (Agarwalla & Sarma, 2016). These innovations would have been of immense benefit during the COVID-19 era had they been taken advantage of earlier.

Evidence suggests that in relation to sign language and AI, there is a plethora of studies conducted for sign language users in different parts of the world; however, currently there is limited research on the application of AI and ML in Africa. A systematic review conducted by Adeyanju et al. (2021) on sign language recognition describes the recent advances in AI and how the advances have paved the way for researchers to apply AI in sign interpretation operations. Specifically, these authors discuss intelligence-based sign language recognition systems, specifically interpretation services, real-time multiperson recognition systems, games, virtual reality environments, natural language communications, online hand tracking of human communication in desktop environments and human–computer interactions (Adeyanju et al., 2021). Save for robotics and virtual reality environments (avatars), as well as hand gestures, none of the listed technologies were gleaned from the studies analysed in the current scoping review. Therefore, there is the potential for creating intelligent solutions to real-time speech-to-text to sign language translation for people with hearing impairment in South Africa. Furthermore, none of the studies in the review conducted real-time captioning in languages other than English.

The results of the review also indicate that there are currently very limited documented studies conducted in South Africa that utilise AI and ML techniques in the translation of South African languages from speech-to-text to sign language, thus highlighting the gap that exists. In the development of a translation tool for South African languages, this current research work will firstly consider the real-time translation of South African languages from speech to text using AI, which may be used, for instance, in creating real-time closed captions that can be used by individuals who have hearing impairments that require assistive technology.

**FIGURE 2:**
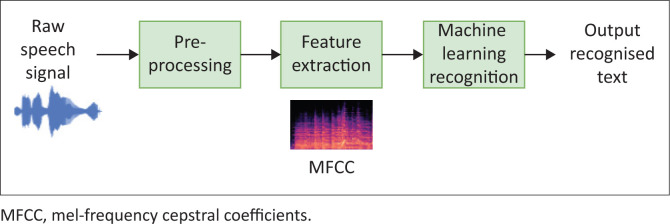
Illustration of the automatic speech recognition process.

South Africa currently has 11 official languages, excluding South African Sign Language, which has recently attained official language status as the twelfth. In South Africa, English is spoken with various accents and is usually mixed with words from Afrikaans and African languages (Lanham, 1996). IsiZulu, isiXhosa, isiNdebele and siSwati, referred to as Nguni languages, have clicking phonemes. Xitsonga has very few clicks, while seSotho, seTswana and Tshivenda are tonal languages (Schulz, Laine, Aunio, & Philippova, 2019). At present, there is limited published evidence on speech-to-text conversion for African languages using AI or ML concepts. Translation of South African English to text with South African accents is not accurately interpreted with the existing Google English-to-text translator. Researchers in South Africa have recently begun to develop tools and applications (apps) to help individuals with hearing impairments. For example, Shezi and Ade-Ibijola (2020) introduced a model and a tool (Deaf Chat) to communicate with hearing-impaired individuals based on AI. Currently, this tool targets the English-speaking South African population. AwezaMed is a recently developed South African app that uses AI for users to pick pairs of languages to translate between (Schwartz, 2020). There thus remains a great need for the development of tools and apps in African languages that can be used for individuals with hearing impairment. These tools and apps must consider (1) the socio-economic factors restricting access to any device or tool that is expensive; and (2) linguistic diversity, multilingualism and their influence on the English accents – and all the unique features of South African languages. Thus, a multilingual speech-to-text translator that is inclusive of all South African languages would make a great contribution to individuals with hearing impairment within this context. To design an end-to-end speech-to-text translator, speech recognition is applied. Speech recognition is a computer science term that refers to a process that converts speech into text (Katyal et al., 2014). Speech recognition is said to be advantageous for people who are hard of hearing, as this technology can be used to convert the speech to text on a computer and other devices (Katyal et al., 2014). Automatic speech recognition systems typically follow the process illustrated in Figure 2.

**FIGURE 3:**
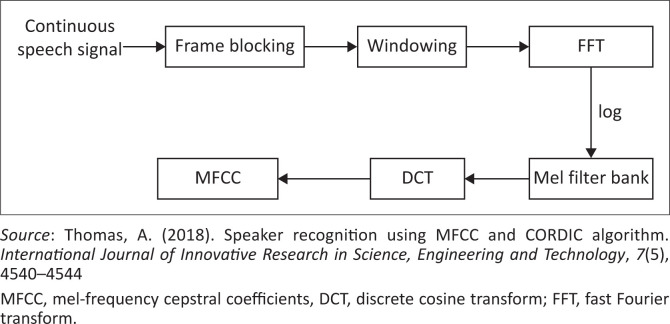
Block diagram for extracting mel-frequency cepstral coefficients.

To translate speech to text for South African languages using ML, huge data sets are required to be used in training the ML model. Therefore, several sources of data sets that could be used in this research work were identified. Firstly, there exist a few corpora for South African languages, including the African Speech Technology Project (Roux, Louw, & Niesler, 2004), the Lwazi project (Badenhorst, Van Heerden, Davel, & Barnard, 2011), the South African corpus of code-switched soap opera speech (Van Der Westhuizen & Niesler, 2018) and the National Centre of Human Language Technology (NCHLT) project (Barnard, Davel, Van Heerden, De Wet, & Badenhorst, 2014). The NCHLT project expands the efforts of the works in Roux et al. (2004) and Badenhorst et al. (2011) to enable the development of large-vocabulary speech recognition systems for practical applications. There are other existing databases, such as the Wall Street Journal (Garofolo, Graff, Paul, & Pallett, 1993), GlobalPhone (Schultz, 2002) and Google (Butryna et al., 2020) corpora. However, these only contain data for the English language. Secondly, the work in Van Der Westhuizen and Niesler (2018) introduced a speech corpus containing 14.3 h of multilingual code-switched speech created from 626 episodes of a South African soap opera. The corpus is divided into four balanced subcorpora, each containing an equal amount of English and Bantu speech: English–isiZulu, English–isiXhosa, English–seTswana and English–seSotho. Code-switched speech refers to the alternation of speech between two or more languages during the discourse. Thirdly, the NCHLT speech corpus contains wide-band prompted monolingual speech from approximately 200 speakers per language, in each of the 11 official South African languages (Barnard et al., 2014). Wikipedia text was used to generate English prompts, while for a few of the languages, a crowd-sourcing approach was followed to generate prompts; for most of the languages, however, prompts were created using text from the South African government website (Barnard et al., 2014). The NCHLT speech corpus contains 50 h of speech per language. Once the data sources have been identified, the first part of ASR, as shown in Figure 2, involves speech pre-processing, which is done to prepare the speech signal for accurate feature extraction. The acoustic environment may have great effects on the generated speech. These include background noises and reverberations that are undesirable. Pre-processing of the speech is, therefore, a necessary step to solve these problems and ultimately improve the accuracy of speech recognition. Speech pre-processing generally involves noise filtration, pre-emphasis, smoothing and echo removing, which could be easily achieved using open-source software such as Audacity. Feature extraction is the next step, which is an integral part of ASR, which aims to reduce the number of features in a data set by selecting prominent features that should ideally retain information regarding the content (speech) while discarding irrelevant information such as speaker accent, speaker identity and environmental conditions like noise and echoing. The features being used can have a significant effect on the performance of the system. Several previous works in ASR have proven mel-frequency cepstral coefficients (MFCC) to be one of the most prominent features of ASR systems. The MFCC features are based on the natural perception of sound in the human ear which makes them suitable for speech recognition. The process of extracting MFCC is shown in Figure 3. The speech frame is passed through a hamming window after pre-processing, and then the energy distribution is calculated using a fast Fourier transform. The effects of harmonics are then eliminated using a mel filter bank, and finally, the discrete cosine transform is applied (Vazhenina & Markov, 2020).

The last step of ASR in Figure 2 entails the application of ML techniques. Some of the ML techniques that have been previously applied and successfully used in the translation of speech to text include artificial neural network (ANN), convolution neural network (CNN) and recurrent neural network (RNN) techniques (Basystiuk et al., 2021; Gibadullin, Perukhin, & Ilin, 2021; Lin, Guo, Zhang, Chen, & Yang, 2020; Kamble, 2016; Morgan, 2011; Passricha & Aggarwal, 2020). An ANN is a collection of algorithms modelled after the human brain for pattern recognition and classification. Neural networks have several advantages, such as reduced computational complexity and the ability to learn any complex relationship between input and output. However, its ‘black-box’ nature can be a disadvantage. Recurrent neural network is derived from ANNs but can learn much faster and effectively. It has feedback connections between its different layers, output–hidden, hidden–output and output–input, forming multiple loops. Some of the loops are in delay form, allowing RNNs to recall past outputs and make selections motivated by what they learned from the past. Convolution neural networks are most prevalent in image processing and computer vision projects. They are made up of filters or kernels which are used to extract relevant features from the input by a convolution operation.

**FIGURE 4:**
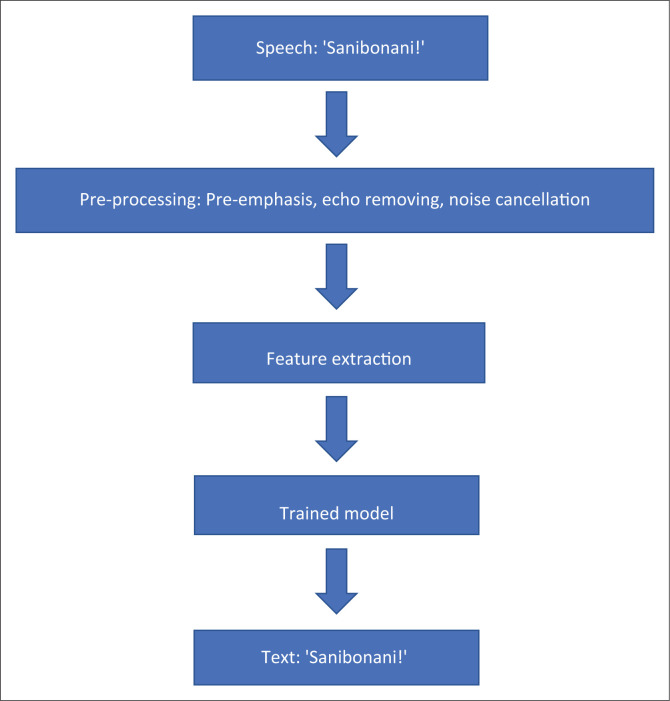
An illustration of the implementation plan and expected results.

To implement the South African speech-to-text translator using ML, the authors of this work propose the use of the NCHLT speech corpus for training as it is a sufficiently large data set, containing approximately 50 h of speech per South African language. The NCHLT contains monolingual speech that allows for the training of the algorithm for each language independently. The data set contains speech prompts in the form of short phrases that will be used to train the ML models. In addition, another data set will be collected from volunteering individuals which will be used for testing the trained models. Three ML architectures will be explored, namely, CNN, RNN and ANN. The models will be trained to recognise the phrases in one of 11 South African languages. The speech signal will be used as input, while the corresponding text will be used as the expected output. Each speech file is annotated with the corresponding text. The data will be split into two, 80% of which will be used for training and 20% for testing. Figure 4 illustrates how a speech signal would be recognised. A raw speech signal is an input to the system, it passes through a pre-processing stage where it undergoes pre-emphasis, echo removing and noises cancellation, and then the key features are extracted into a feature vector. The features are then used as input to the trained ML model which performs pattern recognition to recognise the speech signal and outputs the corresponding text. In Figure 4, the Zulu greeting ‘Sanibonani*!*’ is input as a speech signal and the corresponding text representing the audio signal is outputted.

## Limitations

- This work has only proposed the approach that will be used to conduct the translation from speech to text, without practically demonstrating its application using collected data. The next publication will demonstrate it using real data, the translation from speech to text for South African languages.
- The work has also not demonstrated how the translation from speech-to-text to sign language and from speech to sign language will be conducted. This will be covered in the publications to follow, as this work forms part of a series.

## Conclusion and recommendations

This article highlights that there is a dearth of evidence in the use of AI and ML applications in Africa, with very limited or no tools for speech-to-text to sign language translations for South African languages using AI and ML. This lagging became heightened during COVID-19 and prompted the current researchers to engage in this study. An implementation plan of speech to text using ML for South African languages has thus been presented. Currently, it is viewed that South Africa and Africa as whole seldom apply AI in the development of their systems, especially systems used for the improvement of the social lives of their citizens (Shezi & Ade-Ibijola, 2020). There is a need for a paradigm shift to inclusion of AI and ML as the country transitions towards the 4IR. Model validation using real speech data has currently not been performed and therefore forms part of future plans, together with speech-to-sign-language and speech-to-text to sign language, which will be in subsequent publications.
